# Dimer rattling mode induced low thermal conductivity in an excellent acoustic conductor

**DOI:** 10.1038/s41467-020-19044-w

**Published:** 2020-10-15

**Authors:** Ji Qi, Baojuan Dong, Zhe Zhang, Zhao Zhang, Yanna Chen, Qiang Zhang, Sergey Danilkin, Xi Chen, Jiaming He, Liangwei Fu, Xiaoming Jiang, Guozhi Chai, Satoshi Hiroi, Koji Ohara, Zongteng Zhang, Weijun Ren, Teng Yang, Jianshi Zhou, Sakata Osami, Jiaqing He, Dehong Yu, Bing Li, Zhidong Zhang

**Affiliations:** 1 Shenyang National Laboratory for Materials Science, Institute of Metal Research, Chinese Academy of Sciences, 72 Wenhua Road, Shenyang, 110016 China; 2 School of Materials Science and Engineering, University of Science and Technology of China, Shenyang, 110016 China; 3 Synchrontron X-ray station at SPring-8, Research Network and Facility Services Division, National Institute for Materials Science (NIMS), 1-1-1 Kouto, Sayo-Cho, Sayo-Gun, Hyogo, 679-5148 Japan; 4 Spallation Neutron Source, Oak Ridge National Laboratory, Oak Ridge, TN 37831 USA; 5 Australian Nuclear Science and Technology Organisation, Locked Bag 2001, Kirrawee, DC NSW 2232 Australia; 6 Department of Mechanical Engineering, University of Texas at Austin, Austin, TX 78712 USA; 7 Department of Electrical and Computer Engineering, University of California, Riverside, CA 92521 USA; 8 Department of Physics, Southern University of Science and Technology, Shenzhen, 518005 China; 9 State Key Laboratory of Structural Chemistry, Fujian Institute of Research on the Structure of Matter, Chinese Academy of Sciences, Fuzhou, Fujian 350002 China; 10 Key Lab for Magnetism and Magnetic Materials of the Ministry of Education, Lanzhou University, Lanzhou, 730000 China; 11 SPring-8, Diffraction and Scattering Division, Japan Synchrotron Radiation Research Institute, 1-1-1 Kouto, Sayo-Cho, Sayo-Gun, Hyogo, 679-5198 Japan

**Keywords:** Thermoelectric devices and materials, Two-dimensional materials

## Abstract

A solid with larger sound speeds usually exhibits higher lattice thermal conductivity. Here, we report an exception that CuP_2_ has a quite large mean sound speed of 4155 m s^−1^, comparable to GaAs, but single crystals show very low lattice thermal conductivity of about 4 W m^−1^ K^−1^ at room temperature, one order of magnitude smaller than GaAs. To understand such a puzzling thermal transport behavior, we have thoroughly investigated the atomic structures and lattice dynamics by combining neutron scattering techniques with first-principles simulations. This compound crystallizes in a layered structure where Cu atoms forming dimers are sandwiched in between P atomic networks. In this work, we reveal that Cu atomic dimers vibrate as a rattling mode with frequency around 11 meV, which is manifested to be remarkably anharmonic and strongly scatters acoustic phonons to achieve the low lattice thermal conductivity.

## Abstract (web summary)

CuP2 has a puzzling thermal transport behavior, with low thermal conductivity but quite large mean sound speeds. Here, the authors conduct a systematical study of the atomic structure and lattice dynamics of CuP2 to reveal the origin, finding a dimer rattling behavior.

## Introduction

**Fig. 1:**
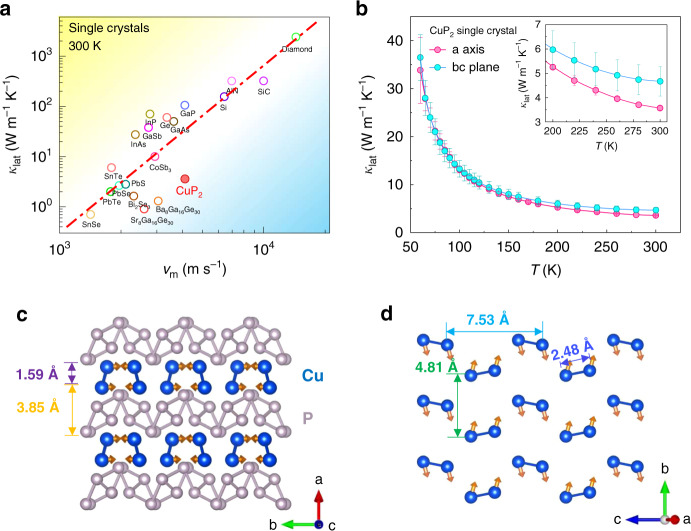
Lattice thermal conductivity and crystal structure of CuP_2_. **a** A survey of lattice thermal conductivity of disorder-free materials vs. mean sound speed *v*_m_. The detailed data are listed in Supplementary Table 1 (for polycrystalline samples, please refer to Supplementary Fig. 1 and Table 2). **b** Temperature dependence of the lattice thermal conductivity of the CuP_2_ single crystal is shown from 50 to 300 K measured by a steady-state comparative method. The inset shows the data near room temperature. The error bars indicate the uncertainty (see “Methods”). **c** The layered structure with Cu dimer layers and P network layers are highlighted. The arrows in orange on Cu atoms represent the vibrational directions at the *Γ* point of the optical phonon mode located at about 11 meV (see Fig. 4). The atomic motions of this mode are also displayed in Supplementary Movie 1. **d** The isolated Cu dimer layers with intra- and inter-dimer distances labeled.

Thermal conduction is one of the most fundamental physical properties of materials^1^. Materials with low thermal conductivity are desirable for a great variety of applications such as thermal insulation^2^, phase transition memory devices^3^, and thermoelectric energy conversion^4^. In electrically insulating nonmagnetic systems, phonons are the major heat carrier, and lattice thermal conductivity is proportional to the product of the square of sound speeds and phonon lifetimes^5–7^. To decrease the thermal conductivity, a general approach is to reduce the phonon lifetimes through enhancing phonon–phonon and phonon-disorder scattering. Phonon–phonon scattering, also known as phonon anharmonic interactions, dominates the thermal conductivity of disorder-free systems like PbTe^8^ and SnSe^9^. There is a special case called rattling, i.e., a localized vibrational mode scattering acoustic phonons with an indication of anti-crossing points in the phonon dispersions^10^. This idea has been broadly applied to rationalize the low thermal conductivity in phonon-glasses thermoelectric materials like filled skutterudites and clathrates^11–13^. In addition, spatially hierarchical atomic disorder has also been widely used, including solid solutions of alloys^14^, nanostructures^15,16^, liquid-like disorder^17,18^ and so forth, to lower the thermal conductivity through phonon-disorder scattering. For a variety of disorder-free compounds, we account for the relationship between thermal conductivity and sound speeds in Fig. 1a (ref. ^19^). In the logarithmic scale, most of the compounds are distributed around an empirical straight line. For a given thermal conductivity value the sound speeds are the dominating factor for materials above the line while the lattice anharmonicity governs thermal transport of materials below the line. Located in the line, single-crystalline diamond^20,21^ has the highest lattice thermal conductivity among those materials, 2400 W m^−1^ K^−1^, accommodating a very high mean sound speed of 14,400 m s^−1^. GaAs is also situated near the line with a mean sound speed of 3627 m s^−1^, whereas CuP_2_ has a similar mean sound speed, but its thermal conductivity is almost ten times lower^22,23^.

The temperature dependencies of lattice thermal conductivity (*κ*_lat_) of CuP_2_ single crystals along *bc* plane and out of *bc* plane are shown in Fig. 1b in the temperature region from 50 to 300 K, respectively. The *κ*_lat_ values in both directions increase as temperature decreases. At 300 K, *κ*_lat_ along the *bc* plane is 4.66 W m^−1^ K^−1^, while 3.57 W m^−1^ K^−1^ out of the *bc* plane. *κ*_lat_ for a polycrystalline sample is shown in Supplementary Fig. 1. At room temperature, it is about 0.62 W m^−1^ K^−1^. This value is greatly reduced compared to the single crystals, which might be attributed to the grain boundaries and defects scattering^24^. In order to understand the mechanism responsible for such a low thermal conductivity in CuP_2_, we conducted a systematical study of the atomic structure and lattice dynamics of this material.

## Results

### Atomic structures

This compound crystallizes in a relatively simple monoclinic structure with space group *P*2_1_/*c* (ref. ^25^). As shown in Fig. 1c, the structure is characteristic of a layered configuration where Cu layers (spacing 1.59 Å) and P network (spacing 3.85 Å) repeat alternatively along the *a*-axis. The Cu atoms forming a dimer occupy an equivalent position. The arrows in orange represent the vibration directions of Cu atoms associated with the rattling mode to be described later. As shown in Fig. 1c, d, the intra-dimer distance is 2.48 Å, while the inter-dimer distances are 3.85, 4.81, and 7.53 Å along *a*, *b*, and *c* directions, respectively. The electron localization function (ELF) calculation was used to support the weak bonding nature between Cu dimers as a direct theoretical evidence (see Supplementary Fig. 2). ELF around Cu atoms is around 0.2, which suggests a weak metallic bond between Cu atoms within Cu dimers, while ELF is almost zero between Cu dimers. This distinction along with the calculated charge density well supports the dimer nature. In consideration of the isolated nature of the Cu dimer, the vibration mode of the dimer as a whole unit can be treated as a localized phonon. Later we will show that these localized phonons play a crucial role in suppressing the thermal conduction of CuP_2_.

**Fig. 2:**
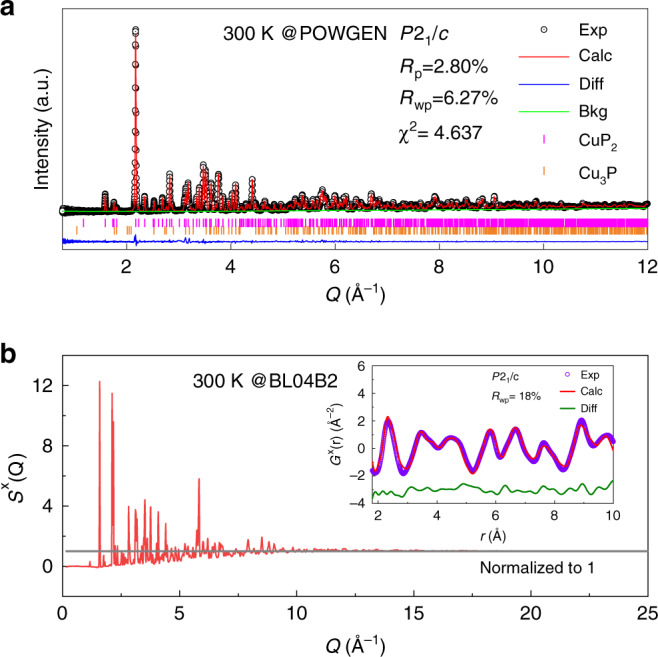
Average and local structures of CuP_2_. **a** Neutron powder diffraction pattern and Rietveld refinement analysis at 300 K. **b** The structure factor, *S*^X^(*Q*), obtained in X-ray total scattering at 300 K. The inset is the reduced PDF, *G*^X^(*r*), as well as the real-space refinement based on the *P*2_1_/*c* crystal model.

Our neutron powder diffraction results confirm the crystal structure as previously reported^25^. Shown in Fig. 2a is the neutron powder diffraction pattern at room temperature (for data at lower temperatures, refer to Supplementary Fig. 3). The Rietveld refinement analysis suggests that the majority phase is monoclinic CuP_2_ while there is a minority phase of Cu_3_P, whose volumetric fraction is only 2.79%. This powder sample, crushed from single crystals, is strongly textured along (100) because of the excellent ductility associated with the layered lattice structure. It is important to notice that the background of the diffraction data is quite flat with no obvious diffuse scattering observed, which indicates no discernable atomic disorder in the sample. The detailed structural parameters determined in the refinements are listed in Supplementary Table 3. The Debye–Waller factors, in particular, *U*_22_ and *U*_33_ of Cu atoms are much larger than those of P atoms. This indicates that there is a significant thermal motion of Cu atoms confined within the layers. The full temperature dependencies of the lattice parameters and Debye–Waller factors are summarized in Supplementary Fig. 4. It should be noted that the fitting of temperature dependencies of Debye–Waller factors to the Einstein model suggests there is negligible atomic disorder. In addition to the powder diffraction, our single-crystal X-ray diffraction also confirms the structure. While the average structural study suggests the right phase and negligible disorder, we further employ the pair distribution function (PDF) analysis to directly confirm the disorder-free nature of the system. As shown in Fig. 2b, the X-ray structure factor *S*^X^(*Q*) is well normalized to 1 at high *Q* at 300 K. Reduced PDF, *G*^X^(*r*), is subsequently obtained via Fourier transform of *S*^X^(*Q*). The inset of Fig. 2b presents the experimental *G*^X^(*r*) that is well reproduced by the *P*2_1_/*c* crystal model, supporting the disorder-free nature.

### Lattice dynamics

**Fig. 3:**
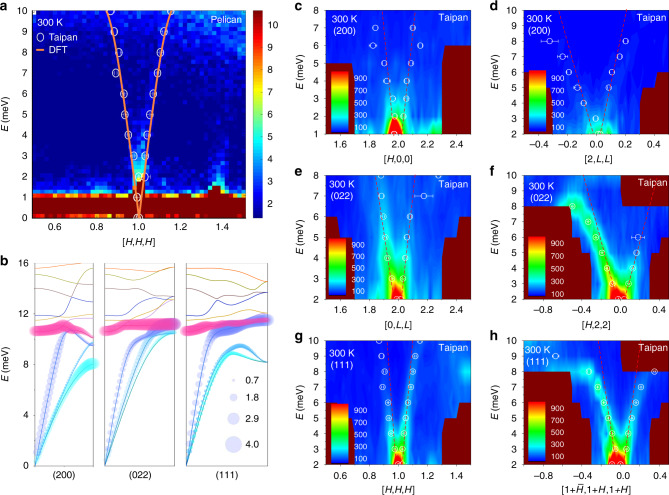
Phonon dispersions of CuP_2_. **a** The phonon dispersion obtained at the time-of-flight neutron spectrometer-Pelican along [*H*, *H*, *H*] direction of Brillouin zone **G** = (111). The circles are the corresponding results obtained from the triple-axis spectrometer—Taipan. The orange lines are the calculated dispersions. **b** Calculated phonon dispersions of Brillouin zones **G** = (200), **G** = (022), and **G** = (111). The size of the shadow bubbles represents the magnitude of Grüneisen parameter (*γ*) of the related branches. **c**–**h** Phonon dispersions of **G** = (200), **G** = (022), and **G** = (111) Brillouin zones collected at Taipan. The circles are the phonon energies determined by the spectral fitting to a Lorentzian function. The red dashed lines represent the linear fitting of phonon branches approaching the Brillouin zone centers to determine the sound speeds.

The above structural study excludes disorder scattering as an origin of the low thermal conductivity. Now, we move to the lattice dynamics study for lattice anharmonicity. We begin with a survey over a large reciprocal space of the dynamic structure function *S*(**Q**, *E*) vs. momentum transfer (**Q**) and energy transfer (*E*) using the time-of-flight neutron spectrometer-Pelican. The measurements are carried out in the scattering plane defined by [*H*, 0, 0] and [0, *L*, *L*]. Shown in Fig. 3a is the phonon dispersion along [*H*, *H*, *H*] direction of the Brillouin zone **G** = (111). The branches of acoustic phonons are clearly observed, in agreement with the results from the triple-axis spectrometer-Taipan and the density functional theory (DFT) calculations. Unfortunately, the optical phonon modes above 10 meV cannot be observed due to limited accessible energy range and energy resolution. For more details of dispersions of different Brillouin zones, please refer to Supplementary Fig. 5. Before we rationally perform detailed INS measurements at Taipan, the lattice dynamics are fully exploited using DFT calculations. The DFT calculated phonon dispersions of Brillouin zones **G** = (200), **G** = (022), and **G** = (111) are plotted in Fig. 3b up to 16 meV while the complete dispersions are plotted in Supplementary Fig. 6. We can see the steep acoustic phonon branches that originate from the individual *Γ* point and reach the zone boundaries at about 10 meV. Above the acoustic phonon branches, there are a few flat optical phonon bands up to 16 meV.

Then, we focus on detailed investigations by conducting constant-*E* as well as constant-**Q** scans for Brillouin zones of **G** = (200), **G** = (022), and **G** = (111) using the thermal neutron triple-axis spectrometer-Taipan in the same scattering plane as on Pelican. The scan directions are summarized in Supplementary Fig. 7. Figure 3c, d shows the dispersions along [*H*, 0, 0] and [0, *L*, *L*] directions of Brillouin zone **G** = (200) at 300 K obtained by constant-*E* scans, respectively. Note that while Fig. 3c represents the longitudinal component of **G** = (200) Brillouin zone, some contributions of the longitudinal phonons are also included in Fig. 3d in addition to the major transverse components (see Supplementary Fig. 7). The phonon intensity decays quickly with departure from the Brillouin zone center. To accurately determine the dispersion relationships, the phonon energies are determined by a spectral fitting to a Lorentzian function. The obtained peak positions are plotted on the contour plots as circles with error bars. Then, we fit the phonon energies to a linear function approaching the zone centers to obtain the experimental sound speeds (see the dash lines). The derived values are listed in Supplementary Table 4. It can be seen that the dispersion along [*H*, 0, 0] direction is much steeper than that along [2, *L*, *L*] direction, indicating larger sound speeds. A similar procedure is applied to the cases of **G** = (022) and **G** = (111), as shown in Fig. 3e–h, respectively.

### Lattice anharmonicity

**Fig. 4:**
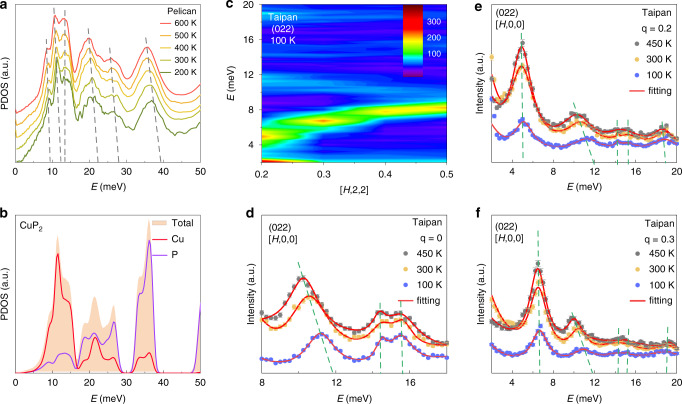
Highly anharmonic optical mode at 11 meV. **a** The experimental PDOS, vertically shifted for clarity. **b**. Calculated total and partial PDOS. **c**. Phonon dispersion of **G** = (022) Brillouin zone along [*H*,0,0] at 100 K. **d**–**f** Temperature dependence of phonon spectra for constant-**Q** scans of **G** = (022) Brillouin zone along [*H*,0,0] with **q** = 0, 0.2, 0.3 at 100, 300 and 450 K, respectively. The green dash lines highlight the energy shift of the peaks at heating.

The temperature-dependent lattice dynamics are considered first on the phonon density of state (PDOS) measurements on a powder sample in a wide temperature region. Shown in Fig. 4a are PDOS at 200, 300, 400, 500, and 600 K, respectively. As temperature rises, it is clear that the whole profile is significantly broadened and several peaks are remarkably softened as an indication of strong anharmonicity. Compared with the DFT calculated PDOS, as plotted in Fig. 4b, it is identified that the Cu atoms mainly participate in the low-energy modes below 15 meV, while the high-energy optical modes are dominated by P atoms. This might be related to the fact that the covalent interactions of P networks are much stronger, in addition to the lighter atomic mass of P than that of the Cu dimer. Of particular interest is the experimentally observed mode located at about 11 meV. Compared with the DFT calculations of the phonon dispersion (Fig. 3b) and PDOS (Fig. 4b), this mode is associated with Cu and has a flat dispersion, thus it must be a rattling mode contributed by the Cu dimer (justified later). The strong anharmonicity of this rattling mode is demonstrated by the large softening from 11.40 meV at 200 K to 10.67 meV at 600 K. Since the acoustic phonons and the nearby low-frequency optical phonons are the main contributors to the thermal conductivity, we further investigate the anharmonic property of this Cu rattling mode in the following sections.

Focusing on the significantly softened mode at about 11 meV, detailed constant-**Q** scans were performed at 100, 300, and 450 K, respectively. Figure 4c shows the dispersion along [*H*, 0, 0] direction of **G** = (022) at 100 K (the results at 300 and 450 K are shown in Supplementary Fig. 8). Just above the intense acoustic phonon branch, a very flat optical phonon branch originates from the Brillouin zone center at about 12 meV to the Brillouin zone boundary at about 10 meV. This is the rattling mode of the Cu dimer. At higher energies, three more flat branches are further observed. The temperature dependencies of these modes are well tracked at the constant-**Q** scans at **q** = 0, 0.2, 0.3, as shown in Fig. 4d–f, respectively. Data at **q** = 0 clearly indicate a distinct behavior for the three optical modes identified. The energy of the first mode (rattling mode) is reduced from 11.16 meV at 100 K to 10.24 meV at 450 K, at a similar rate of −0.002 meV K^−1^ as observed with the PDOS results. In contrast, the other two modes at 14.53 and 15.53 meV at 100 K hardly shift with increasing temperature. At **q** = 0.2 and 0.3 shown in Fig. 4e, f, it is further noticed that the acoustic mode around 6 meV shows no noticeable shift in energy upon temperature increase in contrast to the rattling mode. This behavior is also shown on more data as presented in Supplementary Figs. 9 and 10 for the Brillouin zone **G** = (022). This feature is in sharp contrast to most materials, such as FeSi^26^, where phonon modes are not selectively softened with increasing temperature.

Apart from the remarkable temperature-induced softening of the rattling mode as discussed above, the strong anharmonicity is also manifested by the huge Grüneisen parameter (*γ*)^27,28^ calculated by DFT. In Fig. 3b, the size of the shadow bubbles represents the magnitude of *γ* of a specific mode. Both the rattling mode and the acoustic modes have very large *γ*. The largest *γ* for the rattling mode is about 3.3 at **q** = 0.5, corresponding to the **G** = (022) Brillouin zone boundary, while *γ* is almost larger than 2 in all reciprocal space covered. In particular, *γ* is strikingly large for the longitudinal acoustic (LA) branches (blue color) near the zone boundaries, where they tend to overlap with the rattling mode. In contrast, the Grüneisen parameter of GaAs is almost less than 1 in the whole temperature region below 300 K (ref. ^29^). As a result, the anharmonicity of CuP_2_ is much stronger than that of GaAs with the same magnitude of sound speed, resulting in the huge difference in the lattice thermal conductivity between the two materials. Moreover, our single-crystal X-ray diffraction measurement also suggests that Cu atoms have substantial third-order Debye–Waller factors, which provides further evidence of the strong anharmonicity of Cu-involved vibrations, as listed in Table 1. The values of *U*_222_ and *U*_333_ are 0.0025(6) and 0.00090(13) Å^3^ while *U*_111_ is as small as 0.0006(4) Å^3^. Such a difference is similar to the second-order Debye–Waller factors plotted in Supplementary Fig. 4d. As compared to Cu, the third-order Debye–Waller factors of P atoms are almost negligible within the error bars.

**Table 1:** Third-order Debye–Waller factors of Cu and P atoms determined in refinements of single-crystal X-ray diffraction data.

*U* (Å^3^)	Cu	P	
	Cu1	P1	P2
*U*_111_	0.0006(4)	0.0001(7)	0.0009(13)
*U*_222_	0.0025(6)	0.0011(9)	0.00021(20)
*U*_333_	0.00090(13)	0.00021(20)	−0.0001(2)

### Rattling mode and thermal conductivity

Henceforward, we discuss the nature of the highly anharmonic rattling mode at 11 meV and its impact on the system. Atomic motions of this mode are plotted at the *Γ* point, as shown in Fig. 1c, d, which involve the vibrations of Cu dimers in between the P layers. The atomic motions are recorded in Supplementary Movie 1 and those of the nearby two modes are also present in Supplementary Movie 2 and Supplementary Movie 3, respectively. The justification of calling the vibration of the Cu dimers a rattling mode is further articulated here. Firstly, the Cu dimers behaving as rattlers have very weak bonding to the others as crystallographically and electronically confirmed above. This is also evidenced by the significant Debye–Waller factors of Cu atoms that represent large-amplitude anharmonic vibrations in *bc* plane, in agreement with the well-known skutterudites and clathrates compounds^10,30,31^. Secondly, the dispersion of this mode is quite flat as the group velocity is smaller than 10 m s^−1^ showing an uncorrelated and localized feature. This is in close analogy to the ideal rattling scenario, appearing as a dispersionless Einstein mode with a constant vibration frequency. Thirdly, the anti-crossing or avoided-crossing of the acoustic mode with the rattling mode is also theoretically predicted in the compound, though not experimentally observed due to experiment limitations, as highlighted in Supplementary Fig. 6. The theory predicted five anti-crossing points located at **q** = 0.27 of **G** = ($${\raise0.5ex\hbox{$\scriptstyle 1$}\kern-0.1em/\kern-0.15em \lower0.25ex\hbox{$\scriptstyle 2$}}{\raise0.5ex\hbox{$\scriptstyle 1$}\kern-0.1em/\kern-0.15em \lower0.25ex\hbox{$\scriptstyle 2$}}0$$), **q** = 0.32 of **G** = ($${\raise0.5ex\hbox{$\scriptstyle 1$}\kern-0.1em/\kern-0.15em \lower0.25ex\hbox{$\scriptstyle 2$}}00$$), **q** = 0.37 of **G** = $$(00{\raise0.5ex\hbox{$\scriptstyle 1$}\kern-0.1em/\kern-0.15em \lower0.25ex\hbox{$\scriptstyle 2$}})$$, **q** = 0.13 of **G** = $$(\overline {{\raise0.5ex\hbox{$\scriptstyle 1$}\kern-0.1em/\kern-0.15em \lower0.25ex\hbox{$\scriptstyle 2$}}} {\raise0.5ex\hbox{$\scriptstyle 1$}\kern-0.1em/\kern-0.15em \lower0.25ex\hbox{$\scriptstyle 2$}}0)$$, and **q** = 0.32 of **G** = $$(\overline {{\raise0.5ex\hbox{$\scriptstyle 1$}\kern-0.1em/\kern-0.15em \lower0.25ex\hbox{$\scriptstyle 2$}}} 00)$$. This behavior also gives rise to the decrease of the group velocity of the acoustic mode around the zone boundaries. This rattling mode differs from the previous ones reported in skutterudites and clathrates systems. These compounds have typical cage-like frames with the heavy atoms filled into the lattice void, whereas CuP_2_ crystallizes in a layered structure with Cu dimer as the rattler^10–13,32^. To distinguish with the conventional rattling mode, we term the behavior of CuP_2_ as dimer rattling.

This rattling mode is expected to play a dominating role in the thermal transport. We have systematically determined the sound speeds both experimentally and theoretically, as summarized in Supplementary Table 4. For example, *v*_LA_ = 6243 m s^−1^ and *v*_TA_ = 3192 m s^−1^ are obtained from DFT calculations for the LA and transverse acoustic (TA) branches of **G** = (200), respectively. This *v*_LA_ value is in excellent agreement with that determined by both fitting the INS experimental phonon dispersions and the Brillouin light scattering spectrum (Supplementary Fig. 11), which are 6364 m s^−1^ and 6275 m s^−1^_,_ respectively. The mean sound speed *v*_m_ = 4155 m s^−1^ is estimated using the equation $3v_{\mathrm{m}}^{ - 3} = v_{{\mathrm{LA}}}^{ - 3} + 2v_{{\mathrm{TA}}}^{ - 3}$ (ref. ^13^) based on the theoretical results. As shown in Fig. 1d, unlike the ordinary materials that are distributed around the empirical line, CuP_2_ stands quite exceptional with the thermal conductivity one order of magnitude lower than GaAs that has a similar mean sound speed as CuP_2_^22,23^.

## Discussion

We have discovered the suppressed thermal transport property of CuP_2_ and established a profound understanding on the fundamental physical mechanism by a comprehensive study on the atomic structures and lattice dynamics through neutron, X-ray scattering techniques, and complementary DFT calculations. This system is manifested to be very anharmonic. The Cu atoms participate in a dimer rattling mode, which strongly scatters the LA phonons and leads to anti-crossing phenomena in the dispersion relationships. It is this mode that dominates the reduced thermal conductivity, counteracting the contribution of large mean sound speeds. The observed dimer rattling behavior in the open layered structures might represent an emerging opportunity to rationally tailor thermal transport properties of solids. The combined excellent acoustic conduction and thermal insulation properties may find CuP_2_ a promising material in some nontrivial applications requiring both excellent mechanical sound transmission (or mechanical rigidity) and heat insulation.

## Methods

### Sample preparation

CuP_2_ single crystals were grown through a flux method^33^. Starting materials of Cu (purity: 99.999%), P (purity: 99.999%), and Sn (purity: 99.999%) in a molar ratio of 1:1:3 were placed in an alumina crucible and then sealed into an evacuated quartz tube. The mixture was placed into a box furnace and heated at 1233 K for 6 h. Then, it was cooled down to 873 K at a rate of 3 K/h. The excessive Cu and Sn eutectic flux were decanted in a centrifuge at 873 K. Single crystals were mechanically cleaved from the ingots. Single crystals were crushed for neutron and X-ray powder diffraction as well as for thermal conductivity measurement where a carbon-coated pellet (*ϕ* = 10 mm) was used.

### **Thermal conductivity measurement**

The total thermal conductivity (*κ*_tot_) of the polycrystalline sample was obtained based on the formula *κ*_tot_ = *DC*_p_*ρ*, where *D* is the thermal diffusion coefficient measured using the laser flash method (LFA457, NETZSCH, Germany), *C*_p_ is the Dulong–Petit specific heat capacity, and *ρ* is the density calculated from the geometrical dimensions and mass. Thermal conductivity of single crystals was measured in the temperature range from about 50 to 300 K using a steady-state comparative method^34^. The bar-shaped CuP_2_ samples with a dimension of about 0.5 × 0.5 × 3 mm^3^ were cut from as-grown crystals. The reference was a rod of constantan alloy with a diameter of 0.5 mm. The differential thermocouple was made of copper and constantan wires. In this method, the thermal conductivity of the sample is obtained by measuring the temperature difference between the heat source and heat sink under a steady heat flow through the bar-shaped sample (consist of CuP_2_ single crystal and the reference). The uncertainty of the steady state comparative method is related to the error in measuring sample dimensions, contact thermal resistance, and heat loss. The uncertainty is usually about 10–20% (ref. ^35^). The total thermal conductivity is expressed as a sum of lattice contribution (*κ*_lat_) and electronic contribution (*κ*_el_) for this compound

1 $$\kappa _{{\mathrm{tot}}}=\kappa _{{\mathrm{lat}}}+\kappa _{{\mathrm{el}}}.$$

The electronic part *κ*_el_ is proportional to the electrical conductivity *σ* through the Wiedemann–Franz relation

2 $$\kappa _{{\mathrm{el}}} = L\sigma T,$$

where *L* is Lorenz number^36^. The lattice thermal conductivity *κ*_lat_ can be estimated by subtracting the electrical contribution *κ*_el_. The electrical conductivity of CuP_2_ is only about 8.09 Ω^−1^ cm^−1^ at 300 K (ref. ^37^). *κ*_el_ of CuP_2_ is about 0.0059 W m^−1^ K^−1^ by assuming *L* = 2.45 W Ω K^−2^ (ref. ^38^). In general, the true Lorenz number *L* for most thermoelectric materials is lower than this value so that the electronic contribution to the thermal conductivity is negligible in this system^39^.

### Neutron powder diffraction

The neutron powder diffraction was performed at the time-of-flight powder diffractometer at the Spallation Neutron Source of Oak Ridge National Laboratory, USA^40^. The powder sample with the mass around 3 g was loaded into a vanadium container of 8 mm diameter and measured in a Powgen Automatic Changer covering the temperature region of 10–300 K. The data were collected with neutrons of central wavelength 0.8 Å. Constant temperature scans were conducted at 10, 50, 100, 200, and 300 K, respectively. GSAS^41^ was used to refine the neutron powder diffraction patterns and the results are summarized in Fig. 2a, Supplementary Figs. 3 and 4 as well as Table 3. The Rietveld method employs a nonlinear least-square method to fit the profile that includes all structural and instrumental parameters^42^. Several figure-of-merits including *R*_p_, *R*_wp_, and *χ*^2^ that mean profile residual (reliability factor), weighted profile residual, and goodness of fit are used to quantify the quality of the fit.

### Synchrotron X-ray total scattering

The high-energy X-ray powder diffraction experiment was carried out at the Beamline BL04B2 of SPring-8, Japan^43^. The CuP_2_ powder was put into a quartz capillary (*ϕ* = 1 mm) and then fixed into the sample holder. An empty capillary was measured as a background. X-ray energy was fixed at 61.4 keV with a Si (220) monochromator. The energy resolutions Δ*E*/*E* was approximately 5 × 10^−3^. A vacuum chamber with a Kapton window was used for minimizing the scattering background. Six point-detectors were arranged horizontally to obtain 2*θ* value up to 49^o^ (*Q* range up to 25 Å^−1^). The real-space refinement was performed using PDFgui^44^.

### Single-crystal X-ray diffraction analysis

The single-crystal X-ray diffraction data were collected at a Pilatus CCD diffractometer equipped with graphite-monochromated Mo–*K*_α_ radiation (*λ* = 0.71073 Å) at 293 K. The crystal structure of CuP_2_ was solved, and three-order atomic thermal displacement factors of all atoms were refined through full-matrix least-square technique on *F*^2^. All of the calculations were performed using Jana2006 (ref. ^45^).

### INS measurements

INS experiments were first performed on the time-of-flight cold-neutron spectrometer-Pelican^46,47^, at the Australian Nuclear Science and Technology Organization (ANSTO), Australia. For the PDOS measurements, a powder sample was mounted in an annular aluminum sample can with 1 mm gap. The sample can was attached to the cold head of a closed-cycle refrigerator which is capable of achieving sample temperature from 1.5 to 800 K. The instrument was aligned for 4.69 Å (3.7 meV) incident neutrons. The resolution at the elastic line was 135 µeV. The intensity of an empty can was subtracted as the background contribution and the data were normalized to a vanadium standard that had the same geometry as the sample can. All data manipulations were performed using the Large Array Manipulation Program (LAMP)^48^. The scattering function *S*(**Q**,*E*), as a function of scattering wave vectors (**Q**) and phonon energy (*E*), were measured on energy gain mode over a wide temperature range and then transformed to a generalized PDOS using formula (3), where *k*_*B*_ is Boltzmann’s constant and *T* is temperature

3 $$g\left( E \right) = {\int} {\frac{E}{{Q^2}}S\left( {Q,E} \right)\left( {1 - e^{ - \frac{E}{{k_BT}}}} \right)} dQ.$$

For phonon dispersion measurements, a single crystal weighted 0.7 g was used and pre-orientated such that the scattering plane was defined by [*H*, 0, 0] and [0, *L*, *L*]. A large reciprocal space was covered by rotating the sample over 100° in a step of 1° around the axis perpendicular to the scattering plane. The incident neutron wavelength is 2.345 Å (14.8 meV), corresponding to the second-order reflection of the Pelican highly oriented pyrolytic graphite monochromators configured at 4.69 Å. The sample temperature was set at 300 K. The data were reduced by LAMP and the whole *S*(**Q**,*E*) was generated using HORACE^49^, which was also used to visualize the phonon dispersions. After the general survey over many Brillouin zones using the Pelican instrument, we focused on several selected Brillouin zones **G** = (200), **G** = (022), and **G** = (111) on the thermal neutron triple-axis spectrometer-Taipan at ANSTO^50,51^. The energies of the incident and scattered neutrons were defined by a double-focused PG (002) monochromator and analyzer. We used the open geometry of the neutron beam with a virtual source width of 10 mm at the beam exit from the reactor and a slit of 20 mm at the detector. The measurements were performed with fixed final neutron energy of 14.87 meV. After the sample, a HOPG filter was placed to remove higher-order reflections from the scattered beam. The sample was aligned in the same scattering plane as in the measurements at Pelican. The phonons were measured along [*H*, 0, 0], [*H*, *H*, *H*], and [0, *L*, *L*] directions. A standard cryofurnace was used to access the temperatures of 100, 300, and 450 K. The experimental data were fitted to a Lorentzian function using PAN of DAVE^52^.

### Brillouin light scattering

Brillouin light scattering measurements were performed at room temperature using a Sandercock-type six-pass tandem Fabre–Perot (TFP-2) as a spectrometer and a 532 nm laser as a light source. It is a well-established technique for measuring sound velocities^53^. In backscattering geometry, LA mode sound velocity (*v*_LA_) can be calculated as

4 $$v_{\mathrm{LA}} = \frac{{f_{\mathrm{B}}\lambda _0}}{{2n}}.$$

From the measured Brillouin frequency shift *f*_B_, using this equation, the refractive index *n* = 4.04 and the laser wavelength *λ*_0_ = 532 nm, the LA-wave velocity was determined to be *v*_LA_ = 6275 m s^−1^.

### DFT calculations

All the calculations in this work were performed using the ab initio DFT as implemented in the VASP code^54^. The projector augmented wave pseudopotentials^55^ and the Perdew–Burke–Ernzerhof^56^ functional within the general gradient approximation were used to take care of electron-ion and inter-electron exchange-correlation interactions, respectively. The wave functions were expanded using plane waves with an energy cutoff of 500 eV, and the electronic energy convergence was set to be 10^−8^ eV. The Brillouin zone of the primitive unit cell was sampled in the *Γ*-centered 8 × 10 × 15 *k*-point mesh for structural optimization until all the atomic force is less than 0.001 eV Å^−1^. The phonon dispersion relation was calculated using Phonopy package^57^ combining with VASP using 2 × 2 × 2 supercell with 96 atoms. The *Γ*-centered 2 × 2 × 2 *q*-point mesh was used. To unravel the chemical bonding nature of the Cu–Cu atomic pair, we also calculated the ELF^58^ and electronic charge transfer Δ*ρ* = *ρ*(CuP_2_) − *ρ*(atom), which is defined as the electron charge density difference between CuP_2_ and the constituent individual atoms.

## Supplementary information

Supplementary Information

Description of Additional Supplementary Files

Supplementary Movie 1

Supplementary Movie 2

Supplementary Movie 3

## Data Availability

Available from the corresponding authors on reasonable request.
