# Supplementary material for: Dimer rattling mode induced low thermal conductivity in an excellent acoustic conductor
Source: Nat Commun. 2020 Oct 15;11:5197. doi: 10.1038/s41467-020-19044-w (PMC7566455; doi:10.1038/s41467-020-19044-w)
Supplement: Supplementary file 1 — Supplementary Information [file 41467_2020_19044_MOESM1_ESM.pdf]

Supplementary Figures and Tables for

Dimer rattling mode induced low thermal conductivity in an excellent acoustic conductor

Ji Qi[1,2], Baojuan Dong[1], Zhe Zhang[1,2], Zhao Zhang[1,2], Yanna Chen[3], Qiang Zhang[4], Sergey Danilkin[5], Xi Chen[6,7], Jiaming He[6], Liangwei Fu[8], Xiaoming Jiang[9], Guozhi Chai[10], Satoshi Hiroi[3], Koji Ohara[11], Zongteng Zhang[1,2], Weijun Ren[1], Teng Yang[1,2], Jianshi Zhou[6], Sakata Osami[3], Jiaqing He[8], Dehong Yu[5*], Bing Li[1,2*], Zhidong Zhang[1,2]

[1]Shenyang National Laboratory for Materials Science, Institute of Metal Research, Chinese Academy of Sciences, 72 Wenhua Road, Shenyang 110016, China.
[2]School of Materials Science and Engineering, University of Science and Technology of China, Shenyang 110016, China.
[3]Synchrontron X-ray station at SPring-8, Research Network and Facility Services Division, National Institute for Materials Science (NIMS), 1-1-1 Kouto, Sayo-Cho, Sayo-gun, Hyogo, 679-5148, Japan.
[4]Spallation Neutron Source, Oak Ridge National Laboratory, Oak Ridge, Tennessee 37831, USA.
[5]Australian Nuclear Science and Technology Organisation, Locked Bag 2001, Kirrawee DC NSW 2232, Australia.
[6]Department of Mechanical Engineering, University of Texas at Austin, Austin, TX 78712, USA.
[7]Department of Electrical and Computer Engineering, University of California, Riverside, CA 92521, USA.
[8]Department of Physics, Southern University of Science and Technology, Shenzhen 518005, China.
[9]State Key Laboratory of Structural Chemistry, Fujian Institute of Research on the Structure of Matter, Chinese Academy of Sciences, Fuzhou, Fujian 350002, China.
[10]Key Lab for Magnetism and Magnetic Materials of the Ministry of Education, Lanzhou University, Lanzhou 730000, China.
[11]SPring-8, Diffraction and Scattering Division, Japan Synchrotron Radiation Research Institute, 1-1-1 Kouto, Sayo-Cho, Sayo-gun, Hyogo, 679-5198, Japan.

**Corresponding email:* bingli@imr.ac.cn or dyu@ansto.gov.au

Supplementary Table 1. The lattice thermal conductivity and sound speeds of some disorder-free single crystals at 300 K (corresponding to **Fig. 1a** in the main text).

**Materials**	$\boldsymbol{v_m}$ **(m s^{-1})**	$\boldsymbol{\kappa_{lat}}$ **(W m^{-1} K^{-1})**	**Refs**
PbTe	1780	2.3	[S1, S2]
PbSe	1960	2.64	[S1, S2]
PbS	2110	2.8	[S1, S2]
SnSe	2364	0.62	[S1]
SnTe	1800	5.77	[S3, S4]
Si	6419	156	[S5, S6]
Ge	3358	60	[S5, S6]
GaAs	3627	50	[S7, S8]
InP	2777	70	[S8, S9]
GaP	4110	77	[S8, S9]
InAs	2354	27.3	[S8, S9]
GaSb	2730	37.8	[S8, S9]
$CoSb_3$	2934	10	[S10, S11]
SiC	156	540	[S12]
Diamond	14400	2400	[S12, S13]
$Sr_8Ga_{16}Ge_{30}$	2600	0.9	[S14]
$Ba_8Ga_{16}Ge_{30}$	3046	1.7	[S15, S16]
Bi_2Se_3	2311	1.63	[S17, S18]
AlN	6980	319	[S19]
**CuP_2 (a axis)**	**4155**	**3.57**	**This work**

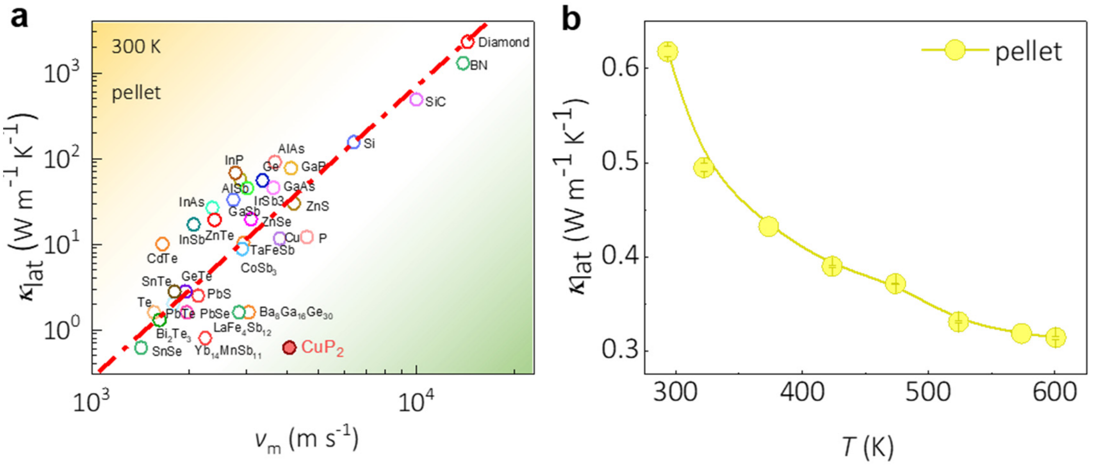

Supplementary Figure 1. Lattice thermal conductivity of polycrystalline samples. a. A survey of lattice thermal conductivity of disorder-free polycrystalline materials versus mean sound speed v_m. **b.** Lattice thermal conductivity of CuP_2 polycrystalline pellet at higher temperatures measured using the thermal diffusion method.

Supplementary Table 2. The lattice thermal conductivity and sound speeds of some disorder-free polycrystalline samples at 300 K (corresponding to Supplementary Figure 1a).

**Materials**	**v_m (m s^{-1})**	**κ_{lat} (W m^{-1} K^{-1})**	**Refs**
PbTe	1783	2	[S2, S20]
PbSe	1963	1.6	[S2, S21]
PbS	2128	2.5	[S2, S22]
GeTe	1944	2.8	[S23]
SnTe	1800	2.8	[S3]
GaP	4110	77	[S8, S24]
GaAs	3627	45.5	[S8, S24]
AlAs	3667	91	[S8, S24]
AlSb	2869	57	[S8, S24]
GaSb	2730	33	[S8, S24]
InP	2777	68	[S8, S24]
InAs	2354	26.5	[S8, S24]
InSb	2062	17	[S8, S24]
ZnS	4193	30	[S8, S25]
ZnSe	3094	19.5	[S8, S25]
ZnTe	2393	19.2	[S8, S25]
CdTe	1651	10	[S8, S25]
Diamond	14400	2300	[S12]
SiC	10000	490	[S12]
Si	6419	156	[S8, S26]
Ge	3358	55.5	[S8]
$Ba_8Ga_{16}Ge_{30}$	3046	1.5	[S16, S27]
Te	1556	1.6	[S28]
Bi_2Te_3	1620	1.3	[S29, S30]
SnSe	2364	7.5	[S31]
$CoSb_3$	2934	10.3	[S32, S33]
$IrSb_3$	3015	45	[S34]
$Yb_{14}MnSb_{11}$	2237	0.8	[S35]
BN(cubic)	13950	1300	[S36]
$LaFe_4Sb_{12}$	2843	1.79	[S37, S38]
TaFeSb	2907	8.8	[S39]
Cu	3796	11.6	[S40, S41]
P(black phosphorus)	4600	12.1	[S42, S43]
**CuP_2**	**4155**	**0.62**	**This work**

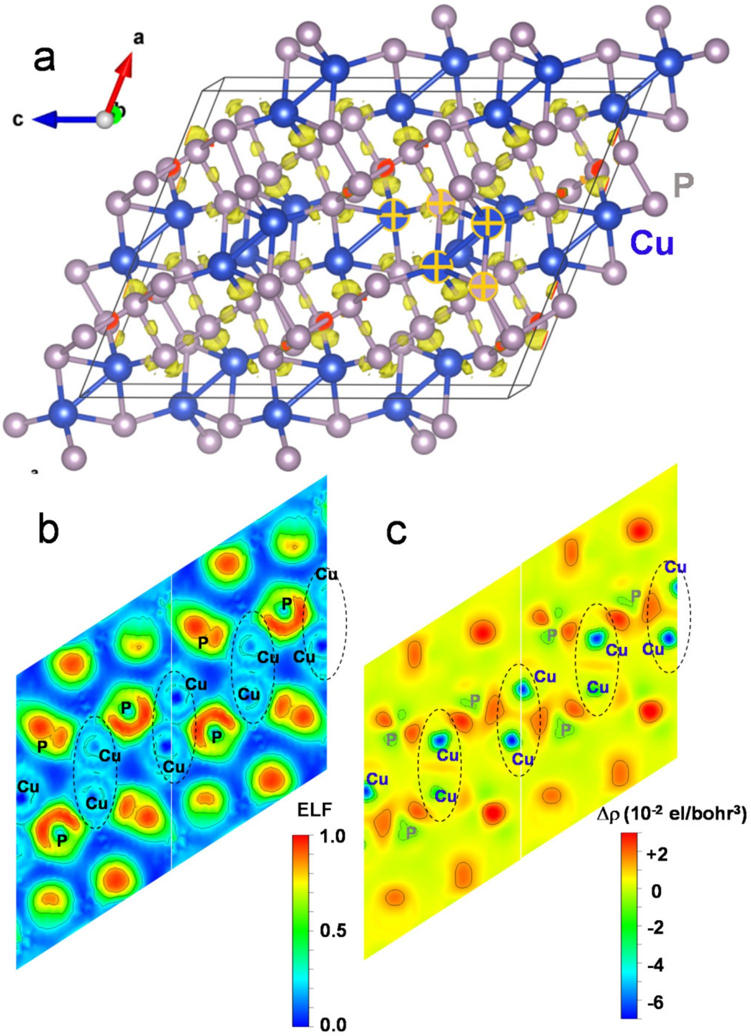

Supplementary Figure 2. The electron localization function (ELF) and the charge density difference Δρ of CuP_2 within the cutting plane ($1\bar{2}3$). a. Atomic structure with the atoms within the cutting plane highlighted by "+". **b.** The ELF. The ELF around Cu atoms is around 0.2 which shows a weak metallic bond between Cu atoms within Cu-Cu dimers. Between Cu-Cu dimers ELF is almost zero, indicating negligible interaction between Cu-Cu dimers. **c.** The charge density difference Δρ. Δρ = ρ(CuP_2) - ρ(atom). Positive and negative values represent electron accumulation and depletion areas, respectively. The charge density in bonding area between the two nearest Cu atoms within Cu-Cu dimers is small, but much larger than that between two nearest Cu-Cu dimers indicate the same results as ELF. Cu-Cu dimers are marked using dash lines.

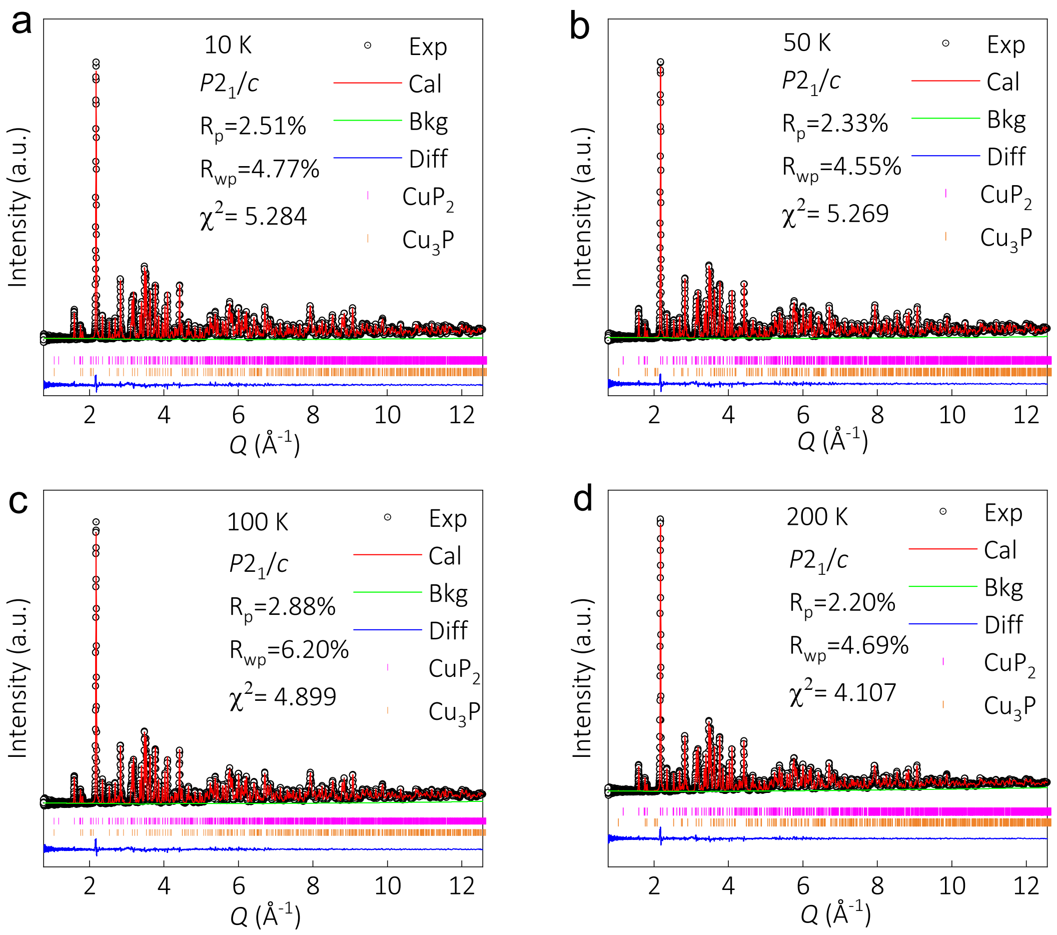

Supplementary Figure 3. Neutron powder diffraction patterns and Rietveld refinements based on the $P2_1/c$ structural model at 10 (a), 50 (b), 100 (c), and 200 K (d).

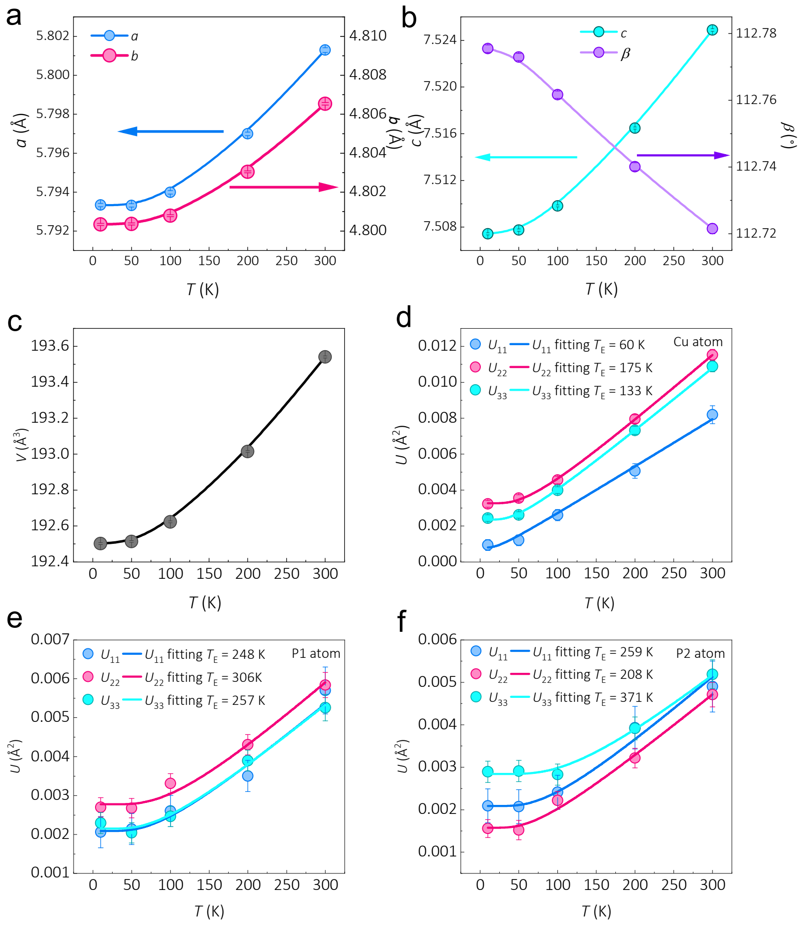

Supplementary Figure 4. Lattice parameters and Debye-Waller factors obtained from Rietveld refinements of neutron diffraction patterns. a-c. Temperature dependencies of lattice parameters a, b, c, angle β, and lattice volume V. **d-f.** Debye-Waller factors U_{11}, U_{22}, and U_{33} of Cu, P1 and P2 atoms, respectively. The solid lines are fitting to the Einstein model[S44]:

$$U = U_{stat} + \frac{\hbar^2}{2k\mu}\frac{1}{T_E}\coth\left(\frac{T_E}{2T}\right). \qquad \text{(S1)}$$

Here, U_{stat} represents the static disorder in the sample crystal structure, the second term is the amplitude of thermal vibrations of atoms. T_E is the Einstein temperature characterizing the interatomic bond stiffness through the effective force constant k_{bond} normalized to the reduced mass μ, defined by $T_E = \sqrt{k_{bond}/\mu}$. ħ is Plank constant and k is Boltzmann constant. The temperature dependencies of Debye-Waller factors of all the atoms in CuP_2 are well fitted to the model with U_{stat} set to zero, which suggests that there is no obvious atomic disorder in this system.

Supplementary Table 3. Crystal structure data at 300 K determined using neutron powder diffraction (corresponding to Fig. 2a).

Temperature	300 K
**Space group**	$P2_1/c$
a **(Å)**	5.80130(12)
b **(Å)**	4.80654(7)
c **(Å)**	7.52489(11)
V **(Å^3)**	193.541(6)
$\alpha = \gamma$ (°)	90
β (°)	112.7215(15)
**Cu coordinate**	(0.14372(19), 0.46100(14), 0.41611(12))
**P1 coordinate**	(0.24960(23), 0.77878(17), 0.69948(14))
**P2 coordinate**	(0.40648(22), 0.11391(17), 0.58022(14))

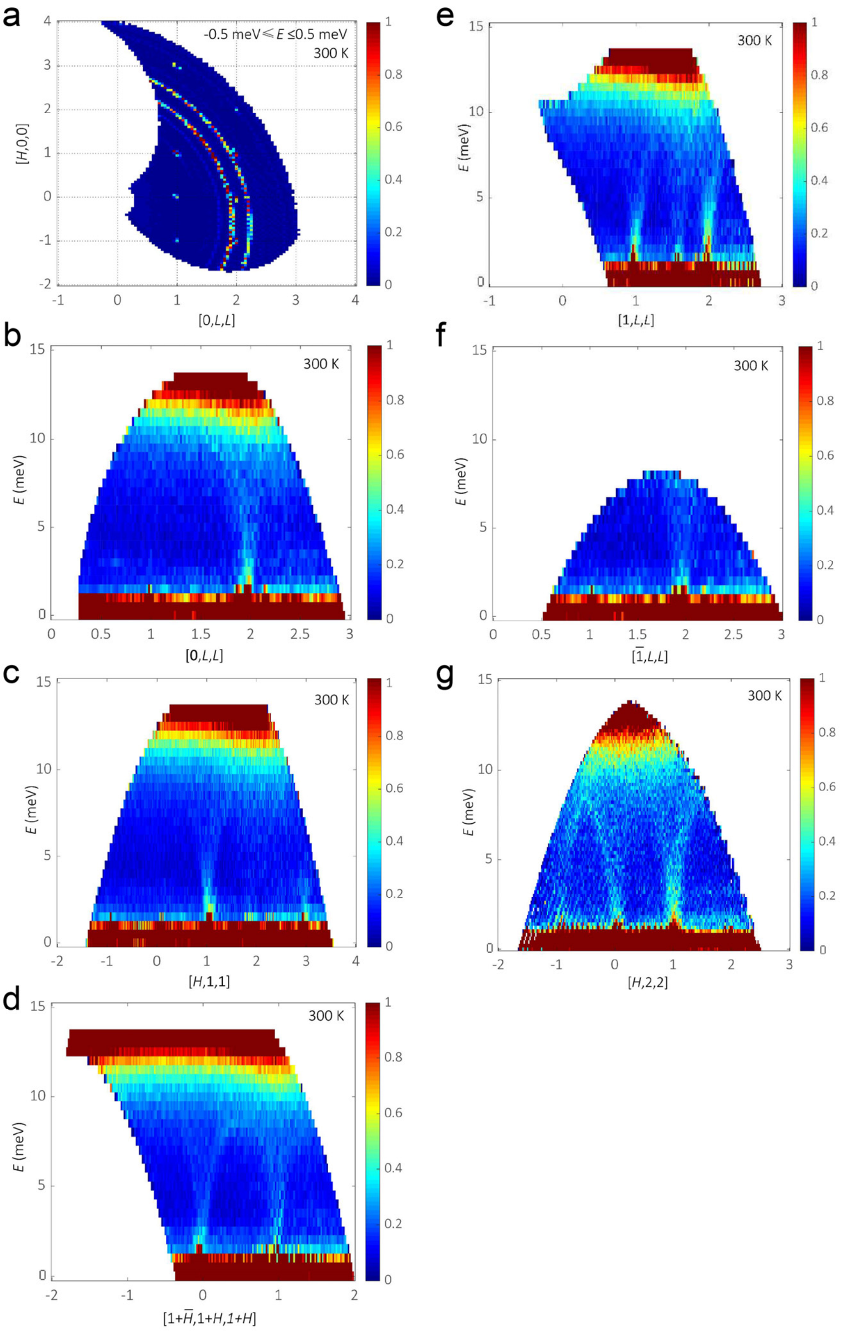

Supplementary Figure 5. Dispersions at several Brillouin zones in the scattering plan defined by [0,*L*,*L*] and [*H*,0,0] measured on Pelican. a. Elastic scattering in this scattering plane. **b**. Dispersion along [0,*L*,*L*]. **c**. Dispersion along [*H*,1,1]. **d**. Dispersion along $[1+\overline{H}, 1+H, 1+H]$. **e**. Dispersion along [1,*L*,*L*]. **f.** Dispersion along $[\overline{1}, L, L]$. **g**. Dispersion along [*H*,2,2].

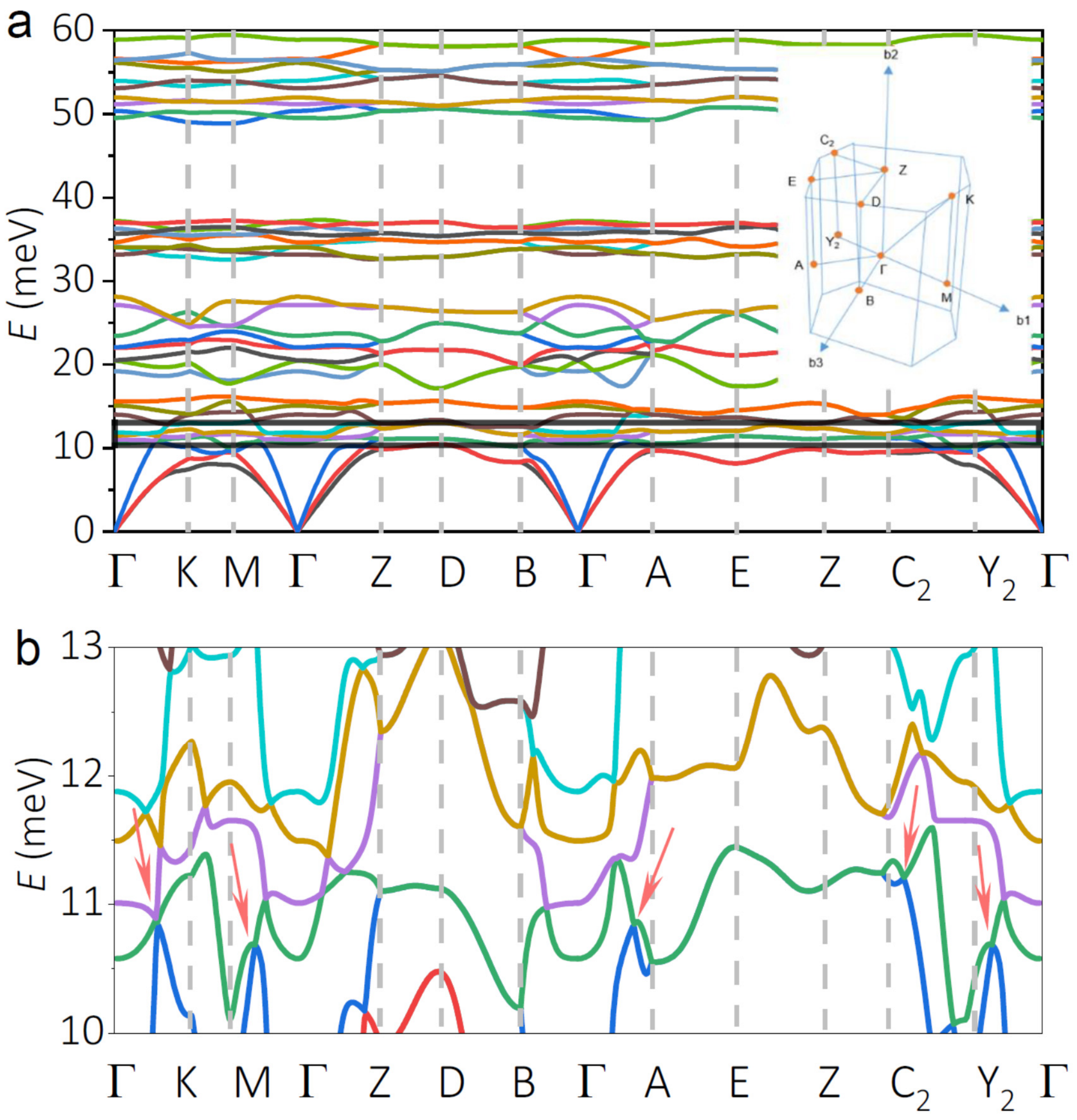

Supplementary Figure 6. DFT calculated phonon dispersions. a. Complete phonon dispersions along K, M, Z, D, B, A, E, C_2, and Y_2 that correspond to $(\frac{1}{2}\frac{1}{2}0)$, $(\frac{1}{2}00)$, $(0\frac{1}{2}0)$, $(0\frac{1}{2}\frac{1}{2})$, $(00\frac{1}{2})$, $(\overline{\frac{1}{2}}0\frac{1}{2})$, $(\overline{\frac{1}{2}}0\overline{\frac{1}{2}})$, $(\overline{\frac{1}{2}}\frac{1}{2}0)$, and $(\overline{\frac{1}{2}}00)$, respectively. The inset is the schematic diagram for the irreducible Brillouin zone setting of CuP_2 with high symmetric *k*-path. **b**. The enlarged region from 10 to 13 meV (labeled by the rectangle in **a**), where the anti-crossing points are marked by red arrows.

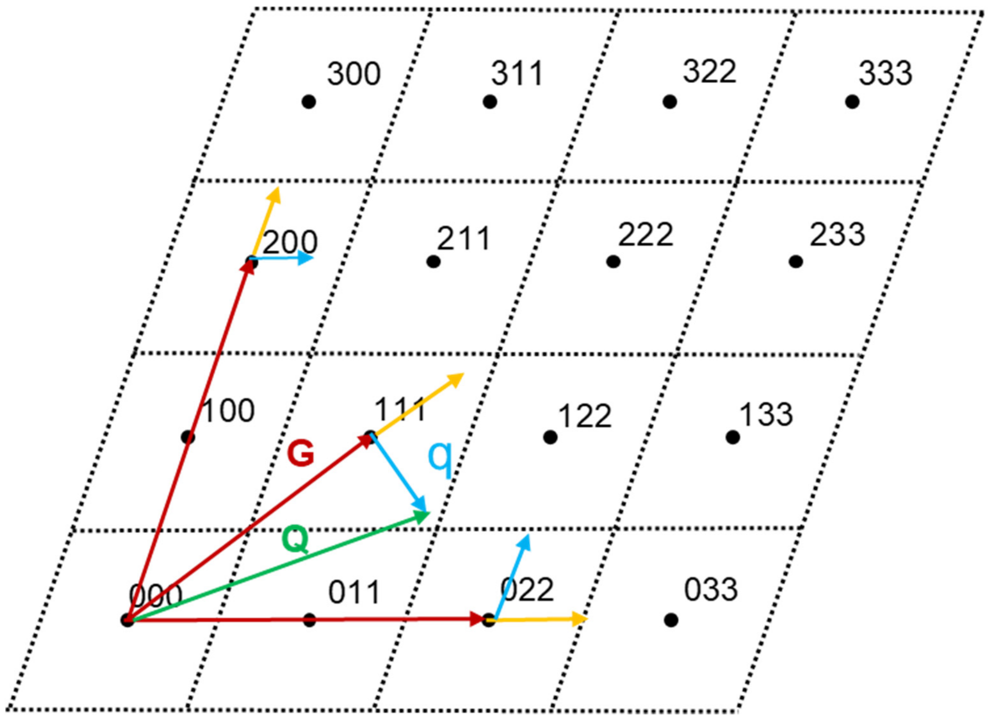

Supplementary Figure 7. Scan summary of INS on Taipan. Reciprocal space is defined by [*H*,0,0] and [0,*L*,*L*] direction. Three Brillouin zones **G** = (200), **G** = (022), and **G** = (111) were accessed. Constant-**Q** scans were performed as the yellow and blue arrows shown. **Q** = **G** + **q** expresses the vector relation due to the momentum conservation [S45]. **G**, **Q,** and **q** represent reciprocal lattice vector, reciprocal scattering vector, and the phonon propagation vector, respectively.

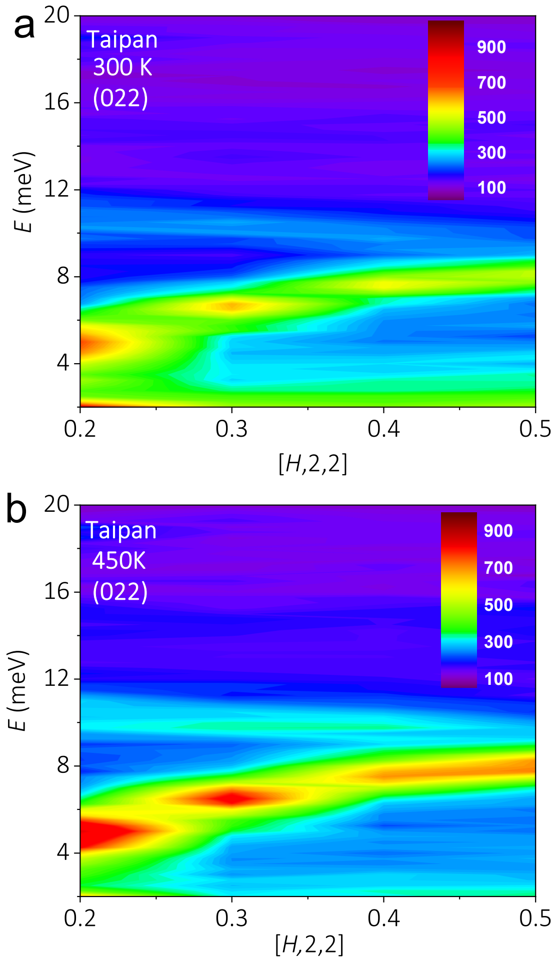

Supplementary Figure 8. Contour plots of constant-Q scans for G = (022) along [*H*,0,0] at 300 (a) and 450 K (b).

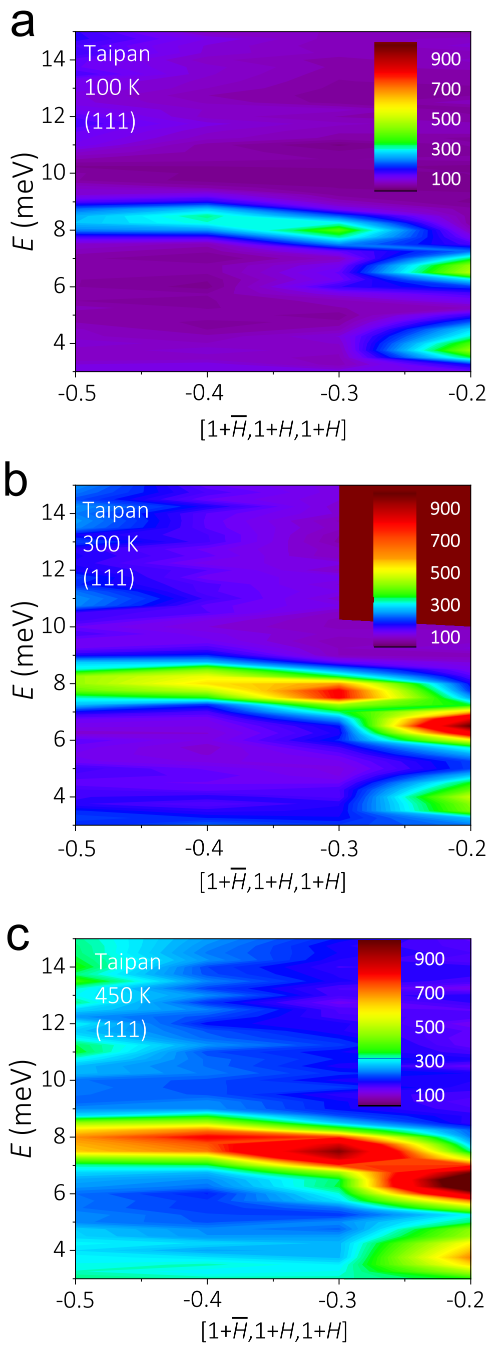

Supplementary Figure 9.Contour plots of constant-Q scans for G = (111) along $[\overline{H}, H, H]$ at 100 (a), 300 (b) and 450 K (c).

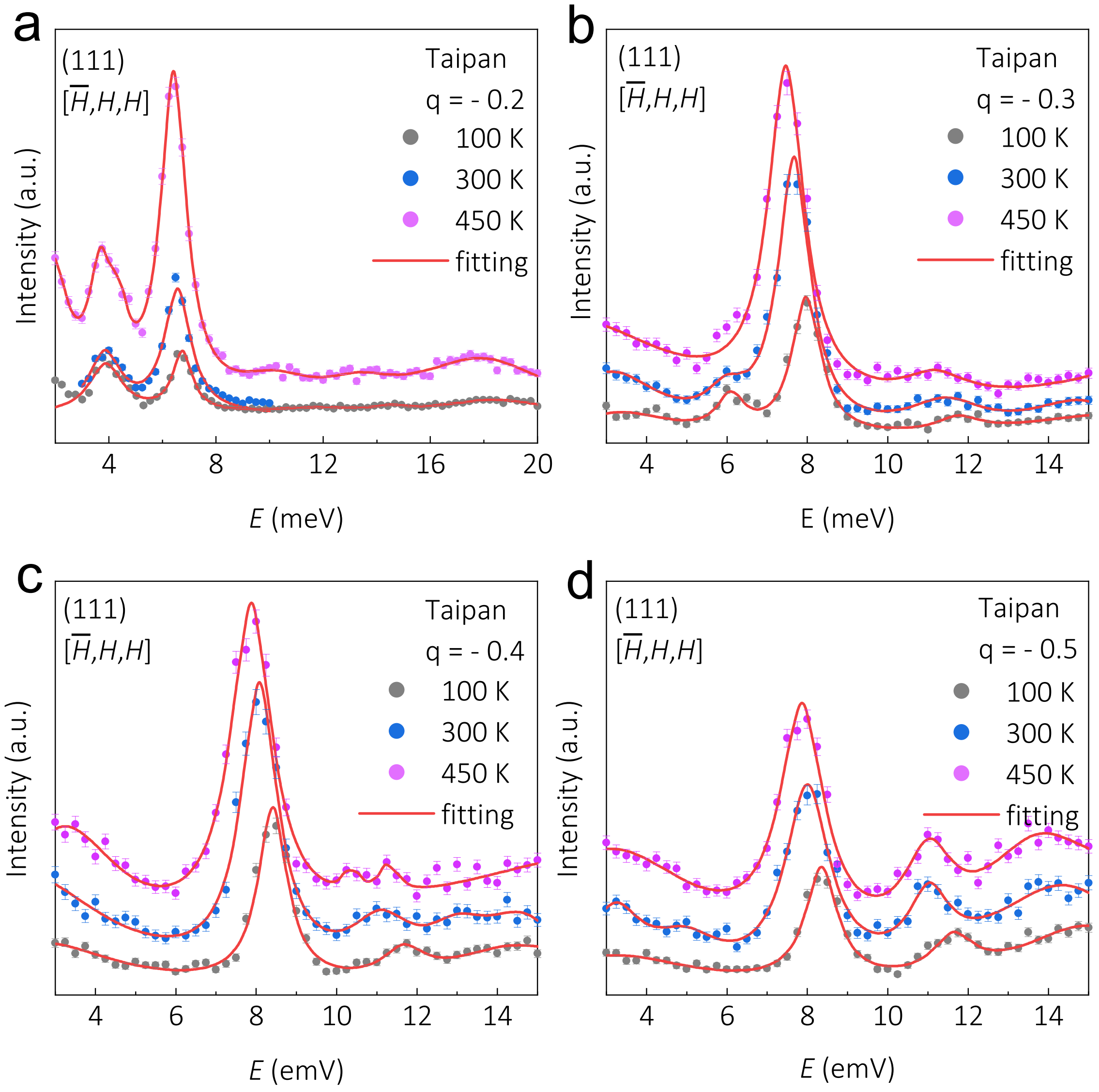

Supplementary Figure 10. Temperature dependencies of constant-Q scans for G = (111) along [$\overline{H}, H, H$] with q = -0.2 (a), -0.3 (b), -0.4 (c) and -0.5 (d) at 100, 300 and 450 K, respectively.

Supplementary Table. 4 Sound speeds of longitudinal acoustic (LA) and transverse acoustic (TA) phonons along different directions determined in several experiments and DFT calculations. The data in the first three columns were determined by fitting the experimental dispersion curves while those in the next three columns were obtained by fitting the calculated dispersion curves. Sound speeds of TA modes were not determined in INS measurements. The calculated mean sound speed of the three Brillouin zones is 4155 m s^{-1}.

Mode	Phonon group velocities (m s^{-1})							
	$(200)_{exp}$	$(022)_{exp}$	$(111)_{exp}$	$(200)_{cal}$	$(022)_{cal}$	$(111)_{cal}$	Brillouin light scattering	Average v_m
v_{LA}	6364	6202	5333	6243	5892	5514	6275	--
v_{TA1}	--	--	--	3192	3558	3568	--	--
v_{TA2}	--	--	--	3095	3101	3131	--	--
v_m	--	--	--	4060	4250	4155	--	4155

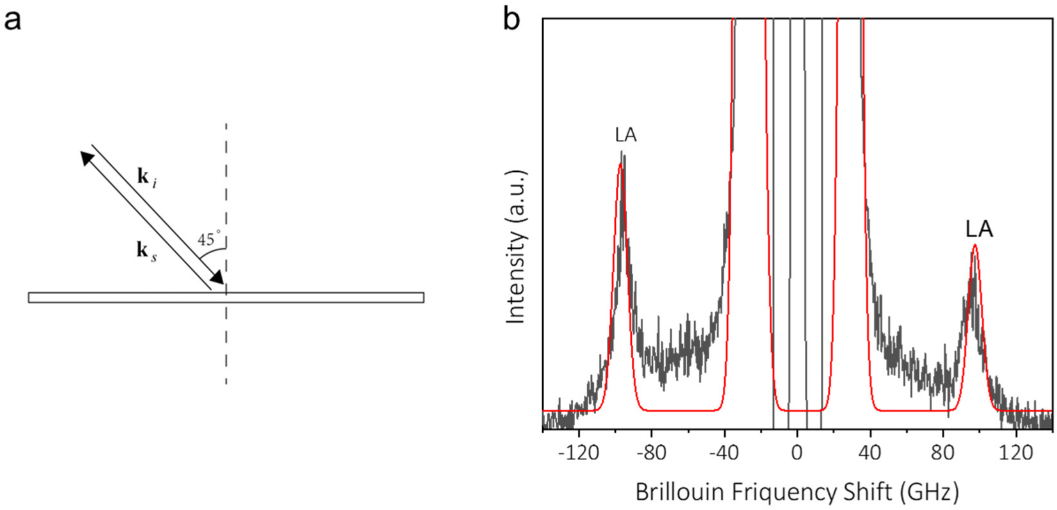

Supplementary Figure 11. Brillouin light scattering. a. The scattering geometry used in the present study. **k**$_i$ and **k**$_s$ are wave vectors of incident and scattering light outside the sample. **b**. The Rayleigh mode and the LA mode are clearly visible on both the stokes and anti-stokes side of the spectra. The LA mode is indicated approximately ±95 GHz in the spectrum.

References

S1. Xiao, Y. et al. Origin of low thermal conductivity in SnSe. *Phys. Rev. B* **94**, 125203 (2016).

S2. Wang, H. et al. High thermoelectric efficiency of n-type PbS. *Adv. Energy Mater.* **3**, 488–495 (2013).

S3. Li, W. et al. Band and scattering tuning for high performance thermoelectric $Sn_{1-x}Mn_xTe$ alloys. *J. Materiomics* **1**, 307–315 (2015).

S4. Bagieva, G. Z. et al. Thermal conductivity of $Sn_{1-x}Mn_xTe$ single crystals. *Inorg. Mater.* **52**, 1215–1219 (2016).

S5. Glassbrenner, C. J. & Slack, G. A. Thermal conductivity of silicon and germanium from 3°K to the melting point. *Phys. Rev* **134**, 1058–1068 (1964).

S6. Phillips. J. C. Vibration spectra and specific heats of diamond-type lattices. *Phys. Rev.* **113**, 147–155 (1959).

S7. Carlson, R. O., Slack, G. A. & Silverman, S. J. Thermal conductivity of GaAs and $GaAs_{1-x}P_x$ laser semiconductors. *J. Appl. Phys.* **36**, 505–507 (1965).

S8. Madelung, O. *Semiconductors: Data Handbook* (Springer Science & Business Media, Marburg, Germany, 2012).

S9. Steigmeier, E. F. & Kudman, I. Thermal conductivity of III-V compounds at high temperatures. *Phys. Rev.* **132**, 508–212 (1963).

S10. Caillat, T., Borshchevsky, A. & Fleurial, J. P. Properties of single crystalline semiconducting $CoSb_3$. *J. Appl. Phys.* **80**, 4442–4449 (1966).

S11. Caillat, T., Borshchevsky, A. & Ferial, J. P. Preparation and thermoelectric properties of p- and n-type $CoSb_3$. *AIP Conference Proceedings.* **316**, 58–61 (1994).

S12. Slack, G. A. Thermal conductivity of pure and impure silicon, silicon carbide, and diamond. *J. Appl. Phys.* **35**, 3460–3466 (1964).

S13. Inyushkin, A. V. & Taldenkov, A. N. Thermal conductivity of high purity synthetic single crystal diamonds. *Phys. Rev. B* **97**, 144305 (2018).

S14. Chakoumakos, B. C. et al. Structural disorder and thermal conductivity of the semiconducting clathrate $Sr_8Ga_{16}Ge_{30}$. *J. Alloy. Compd.* **296**, 80–86 (2000).

S15. Sales, B. C. et al. Structural, magnetic, thermal, and transport properties of $X_8Ga_{16}Ge_{30}$ (X = Eu, Sr, Ba) single crystals. *Phys. Rev. B* **63**, 254113 (2001).

S16. Toberer, E. S. et al. High temperature thermoelectric efficiency in $Ba_8Ga_{16}Ge_{30}$ *Phys. Rev. B* **77**, 075203 (2008).

S17. Navrátil, J. et al. Transport properties of $Bi_{2-x}In_xSe3$ single crystals. *J. Solid State Chem.* **160**, 474–481 (2001).

S18. Xiang, G. et al. First-principles study of structural, elastic, electronic and thermodynamic properties of topological insulator Bi_2Se_3 under pressure. *Philos. Mag.* **96**, 6435–6443 (2016).

S19. Slack, G. A. et al. The intrinsic thermal conductivity of AlN. *J .Phys. Chem. Solids* **48**, 641–647 (1987).

S20. Pei, Y. et al. High thermoelectric performance in PbTe due to large nanoscale Ag_2Te precipitates and La doping. *Adv. Funct. Mater.* **21**, 241–249 (2011).

S21. Wang, H. et al. Weak electron-phonon coupling contributing to high thermoelectric performance in n-type PbSe. *Proc. Natl. Acad. Sci. U.S.A.* **109**, 9705–9709 (2012).

S22. Zhao, L. et al. Thermoelecrics with earth abundant elements: high performance p-type PbS Nanostructured with SrS and CaS. *J. Am. Chem. Soc.* **134**, 7902–7012 (2012).

S23. Gelbstein, Y. et al. Significant lattice thermal conductivity reduction following phase separation of the highly efficient $Ge_xPb_{1-x}Te$ thermoelectric alloys. *Phys. Status Solidi B* **7***,* 1431–1437 (2014).

S24. Nakwaski, W. O. Thermal conductivity of binary, ternary, and quaternary IIIV compounds. *J. Appl. Phys.* **64**, 159–166 (1988).

S25. Bijalwan, R. D., Rami, P. N., & Tiwarii, M. D. Lattice thermal conductivity of II-VI compounds. *J. Phys. C: Solid State Phys.* **16**, 2537–2545 (1983).

S26. Bux, S. K. et al. Nanostructured bulk silicon as an effective thermoelectric material. *Adv. Funct. Mater.* **19**, 2445–2452 (2009).

S27. Toberer, E. S. et al. High temperature thermoelectric efficiency in $Ba_8Ga_{16}Ge_{30}$. *Phys. Rev. B* **77**, 075203 (2008).

S28. Sun, L. et al. Tellurium as a high performance elemental thermoelectric. *Nat.*

Commun. **7**, 10287 (2016).

S29. Qiu, B., Sun, L. & Ruan, X. L. Lattice thermal conductivity reduction in Bi_2Te_3 quantum wires with smooth and rough surfaces: A molecular dynamics study. *Phys. Rev. B* **83**, 035312 (2001).

S30. Zhang, Q. et al. Improved thermoelectric performance of silver nanoparticles-dispersed Bi_2Te_3 composites deriving from hierarchical two-phased heterostructure. *Adv. Funct. Mater.* **25**, 966–976 (2015).

S31. Xiao, Y. et al. Origin of low thermal conductivity of SnSe. *Phys. Rev. B* **94**, 125203 (2016).

S32. Caillat, T., Borshchevsky, A. & Ferial, J. P. Preparation and thermoelectric properties of p- and n-type $CoSb_3$. *AIP Conference Proceedings.* **316**, 58-61 (1994).

S33. Shi, X. et al. Multiple-filled skutterudites: High thermoelectric figure of merit through separately optimizing electrical and thermal transports. *J. Am. Chem. Soc.* **133**, 7837–7846 (2011).

S34. Slack, G.A., & Tsoukala, V. G. Some properties of semiconducting $IrSb_3$. *J. Appl. Phys.* **76**, 1665–1671 (1994).

S35. Brown, S. R. et al. $Yb_{14}MnSb_{11}$: New high efficiency thermoelectric materials for power Generation. *Chem. Mater.* **18**, 1873–1877 (2006).

S36. Slack, G. A. Nonmetallic crystals with high thermal conductivity. *J. Phys. Chem. Solid.* **34**, 321–335 (1973).

S37. Koza, M. M. et al. Vibrational dynamics of filled skutterudites LaT_4X_{12} (T = Fe, Ru, Os, X = As, Sb). *J. Phys. Soc. Jpn*. **82**, 11467 (2013).

S38. Qiu, P. F. et al. High-temperature electrical and thermal transport properties of fully filled skutterudites RFe_4Sb_{12} (R = Ca, Sr, Ba, La, Ce, Pr, Nd, Eu, and Yb). *J. Appl. Phys.* **109**, 63713 (2011).

S39. Zhu, H. et al. Discovery of TaFeSb-based half-Heuslers with high thermoelectric performance. *Nat. Commun.* **10**, 270 (2019).

S40. Moore, J. P., Mcelroy, D. L. & Graves, R. S. Thermal conductivity and electrical resistivity of high-purity copper from 78 to 400 °K. *Can. J. Phys.* **45**, 3849–3865 (1967).

S41. Ledbetter, H. M. Sound velocities and elastic-constant averaging for polycrystalline copper. *J. Phys. D: Appl. Phys.* **13**, 1879–1884 (1980).

S42. Kaneta, C., Katayama-Yoshida, H. & Morita, A. Lattice dynamics of black phosphorus. *Solid State Commun.* **44**, 613–617 (1982).

S43. Slack, G. A. Thermal conductivity of elements with complex lattices: B, P, S. *Phys. Rev.* **139**, 507–515 (1965).

S44. Sevilano, E., Meuth, H. & Rehr, J. J. Extend x-ray absorption fine structure Debye-Waller factors. I. Monatomic crystals. *Phys. Rev. B* **20**, 4908–4911 (1979).

S45. Shirane, G., Shapiro, S. M. & Tranquada, J. M. *Neutron Scattering with a Triple-Axis Spectrometer: Basic Techniques* (Cambridge University Press, Cambridge, UK, 2002).
