# Supplementary material for: Dimer rattling mode induced low thermal conductivity in an excellent acoustic conductor
Source: Nat Commun. 2020 Oct 15;11:5197. doi: 10.1038/s41467-020-19044-w (PMC7566455; doi:10.1038/s41467-020-19044-w)
Supplement: Supplementary file 2 — Description of Additional Supplementary Files [file 41467_2020_19044_MOESM2_ESM.pdf]

## Description of Additional Supplementary Files

Supplementary Movie 1: Atomic motions of 4th branch at 10.58meV

Supplementary Movie 2: Atomic motions of 5th branch at 11.01meV

Supplementary Movie 3: Atomic motions of 6th branch at 11.49meV
